# The impact of COVID-19 on college students’ physical activity: A protocol for systematic review and meta-analysis

**DOI:** 10.1097/MD.0000000000027111

**Published:** 2021-09-03

**Authors:** Yunxia Ding, Song Ding, Jiali Niu

**Affiliations:** a Sports Industry and Leisure College, Nanjing Sport Institute, Jiangsu, China; b Department of Infection, Jingjiang People's Hospital, the Seventh Affiliated Hospital of Yangzhou University, Jiangsu, China; c Department of Pharmacy, Jingjiang People's Hospital, the Seventh Affiliated Hospital of Yangzhou University, Jiangsu, China.

**Keywords:** college students, coronavirus disease 2019, meta-analysis, physical activity

## Abstract

**Background:**

We aimed to conduct a meta-analysis to assess the impact of coronavirus disease 2019 (COVID-19) on college students’ physical activity.

**Methods:**

All cohort studies comparing college students undertaking physical exercise at school before the COVID-19 pandemic and physical exercise at home during the COVID-19 pandemic will be included in this review. We will use index words related to college students, physical exercise, and COVID-19 to perform literature searches in the PubMed, Medline, Embase, and CNKI databases, to include articles indexed as of June 20, 2021, in English and Chinese. Two reviewers will independently select trials for inclusion, assess trial quality, and extract information for each trial. The primary outcomes are exercise frequency, duration, intensity, and associated factors. Based on the Cochrane assessment tool, we will evaluate the risk of bias of the included studies. Revman 5.3 (the Cochrane collaboration, Oxford, UK) will be used for heterogeneity assessment, data synthesis, subgroup analysis, sensitivity analysis, and funnel plot generation.

**Result:**

We will discuss the impact of COVID-19 on college students’ physical activity.

**Conclusion:**

Stronger evidence about the impact of COVID-19 on college students’ physical activity will be provided to better guide teaching practice.

**Systematic review registration:**

PROSPERO CRD42021262390.

## 1 Introduction

The coronavirus disease 2019 (COVID-19) is a global pandemic^[1]^ leading to a global shutdown that closed schools for months.^[2,3]^ In many nations, schools were closed to students,^[4]^ and teachers directed educational activities remotely via digital devices or homeschooling resources.^[5]^ However, in contrast to exercising at school, home exercise during the COVID-19 pandemic was affected by various factors,^[6,7]^ such as limited venues, family sports atmosphere, and incomplete equipment, it was difficult for college students to reach school requirements.^[8–11]^

To promote college students’ active participation in physical exercise and provide reference for teaching practice, we aim to conduct a meta-analysis of cohort studies to assess the impact of COVID-19 on college students’ physical activity.

## 2 Methods

### 2.1 Registration

This protocol was registered on the International Prospective Register of Systematic Reviews on July 1 as CRD42021262390. In this paper, we will perform the protocol according to the Cochrane Handbook for Systematic Reviews of Interventions and preferred reporting items for systematic review and meta-analysis protocols guidelines.^[12,13]^

### 2.2 Inclusion criteria for considering studies

#### 2.2.1 Types of studies

All cohort studies comparing college students undertaking physical exercise at school before the COVID-19 pandemic and physical exercise at home during the COVID-19 pandemic will be included in this review.

#### 2.2.2 Types of participants

College students, grade 1 to 4.

#### 2.2.3 Types of interventions

The impact of COVID-19 on college students’ physical activity.

#### 2.2.4 Types of outcome assessments

Any available information about the impact of COVID-19 on college students’ physical activities will be assessed. The primary outcomes are exercise frequency, duration, intensity, and associated factors.

### 2.3 Search strategy

We will use index words related to college students’ physical activity and COVID-19 to perform literature searches in the PubMed, Embase, Medline, and CNKI databases, to include articles indexed as of June 20, 2021, in English and Chinese. The key search terms will be used are [“Exercises” OR “Physical Activity” OR “Activities, Physical” OR “Activity, Physical” OR “Physical Activities” OR “Exercise, Physical” OR “Exercises, Physical” OR “Physical Exercise” OR “Physical Exercises” OR “Acute Exercise” OR “Acute Exercises” OR “Exercise, Acute” OR “Exercises, Acute” OR “Exercise, Isometric” OR “Exercises, Isometric” OR “Isometric Exercises” OR “Isometric Exercise” OR “Exercise, Aerobic” OR “Aerobic Exercise” OR “Aerobic Exercises” OR “Exercises, Aerobic” OR “Exercise Training” OR “Exercise Trainings” OR “Training, Exercise” OR “Trainings, Exercise” AND “2019 novel coronavirus disease” OR “COVID19” OR “COVID-19 pandemic” OR “SARS-CoV-2 infection” OR “COVID-19 virus disease” OR “2019 novel coronavirus infection” OR “2019-nCoV infection” OR “coronavirus disease 2019” OR “coronavirus disease-19” OR “2019-nCoV disease” OR “COVID-19 virus infection”].

### 2.4 Data collection

#### 2.4.1 Selection of studies

**Figure 1:**
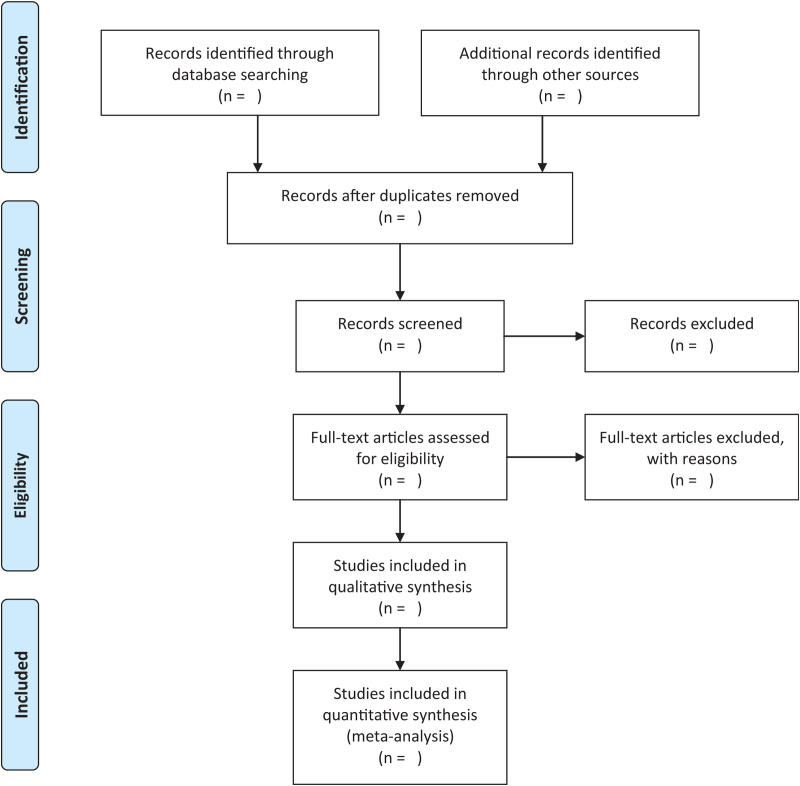
Flow diagram of the study selection process. Moher D, Liberati A, Tetzlaff J, Altman DG, The PRISMA Group. 2009. *P*referred *R*eporting *I*tems for *S*ystematic Reviews and *M*eta-*A*nalyses: The PRISMA Statement. PLoS Med 6(6): e1000097. doi:10.1371/journal.pmed1000097.

Two reviewers will independently select trials for inclusion. Articles will be excluded if they meet any of the following criteria: the object is not a college student, fewer than 10 students, and studies not comparing college students undertaking physical exercise at school before the COVID-19 pandemic undertaking physical exercise at home during COVID-19 pandemic. The study selection process is illustrated in Figure 1.

#### 2.4.2 Data and information extraction

Two authors will extract general information independently for each included trial, including the name of the first author, year, country, design, sample size, average age, and sex ratio. The third author will check all the data.

In the same manner, we will extract the data for impact assessments. For each study, we will extract the following information: exercise frequency, duration, intensity, and associated factors. We will resolve the disagreements through discussion.

### 2.5 Assessment of risk of bias

The review authors will independently assess the quality of the trials included in the review, in accordance with Chapter 8 of the Cochrane Handbook for Systematic Reviews of Interventions (Higgins 2011), by allocation concealment (selection bias); blinding (performance bias and detection bias); blinding of participants and personnel (performance bias); blinding of outcome assessment (detection bias); incomplete outcome data (attrition bias); selective reporting (reporting bias); and other bias. The fifth author will check all the data. We will use this information to evaluate quality and resolve disagreements by discussion until a consensus is reached.

### 2.6 Data analysis

#### 2.6.1 Assessment of heterogeneity

We will use chi-square test and *I*^2^ statistic to assess heterogeneity. If the heterogeneity is within the acceptable range (*P* > .10, *I*^2^ < 50%), the fixed effects model shall be used for data analysis; otherwise, the random effects model will be used.

#### 2.6.2 Date synthesis

Two authors will extract information independently for each trial. The third author will check all the data. Review Manager 5.3 (the Cochrane collaboration, Oxford, UK) will be used to assess the risk of bias, heterogeneity, sensitivity, and subgroup analysis. We will calculate a weighted estimate across trials and interpret of the results. Statistical significance will be set at *P* < .05.

#### 2.6.3 Subgroup analysis

We will perform the following subgroup analysis to explore the possible causes of high heterogeneity: grade (1, 2, 3, and 4), gender (male and female), and different counties.

#### 2.6.4 Sensitivity analysis

Sensitivity analysis will be conducted by excluding trials one by one and observing whether the synthesis result changes significantly. If there are significant changes, we will cautiously make a decision to decide whether to merge them. If there is little change, this indicates that our synthesized result is firm.

### 2.7 Assessment of publication bias

If more than 10 articles are available for analysis, funnel plots will be generated to assess publication bias. A symmetrical distribution of funnel plot data indicates that there is no publication bias; otherwise, we will analyze the potential reasons for this outcome and provide a reasonable interpretation for asymmetric funnel plots.

### 2.8 Confidence in cumulative evidence

The Grades of Recommendations Assessment, Development and Evaluation system will be used to assess the quality of our evidence.^[14]^ According to the grading system, the level of evidence will be rated as high, moderate, low, and very low.

## 3 Discussion

COVID-19 is an emerging, rapidly evolving situation that leads to global shutdown.^[15]^ Schools were closed for months, and students took online courses at home.^[16,17]^ It was very important to pay attention to the physical and mental health of students and to guide students in strengthening exercises. Teachers encouraged students to perform physical exercise through various methods, such as online physical education, assigning exercise assignments, and cloud competitions. However, home physical exercises rely more on students’ independent practices, and teachers lack effective monitoring.^[18]^ The physical exercise of college students at home may not meet the standards.^[19,20]^

By conducting a meta-analysis of related cohort studies, we will provide the impact of COVID-19 on college students’ physical activity to better guide teaching practice.

## Author contributions

**Conceptualization:** Yunxia Ding, Song Ding, Jiali Niu.

**Investigation:** Yunxia Ding, Song Ding, Jiali Niu.

**Methodology:** Yunxia Ding, Song Ding, Jiali Niu.

**Software:** Yunxia Ding, Jiali Niu.

**Supervision:** Yunxia Ding, Song Ding, Jiali Niu.

**Writing – original draft:** Yunxia Ding, Song Ding, Jiali Niu.

**Writing – review & editing:** Yunxia Ding, Song Ding, Jiali Niu.
